# Supplementary material for: Extracellular Vesicles from SOD3-Transduced Stem Cells Exhibit Improved Immunomodulatory Abilities in the Murine Dermatitis Model
Source: Antioxidants (Basel). 2020 Nov 23;9(11):1165. doi: 10.3390/antiox9111165 (PMC7700433; doi:10.3390/antiox9111165)
Supplement: Supplementary file 1 [file antioxidants-09-01165-s001.pdf]

Supplementary Figures

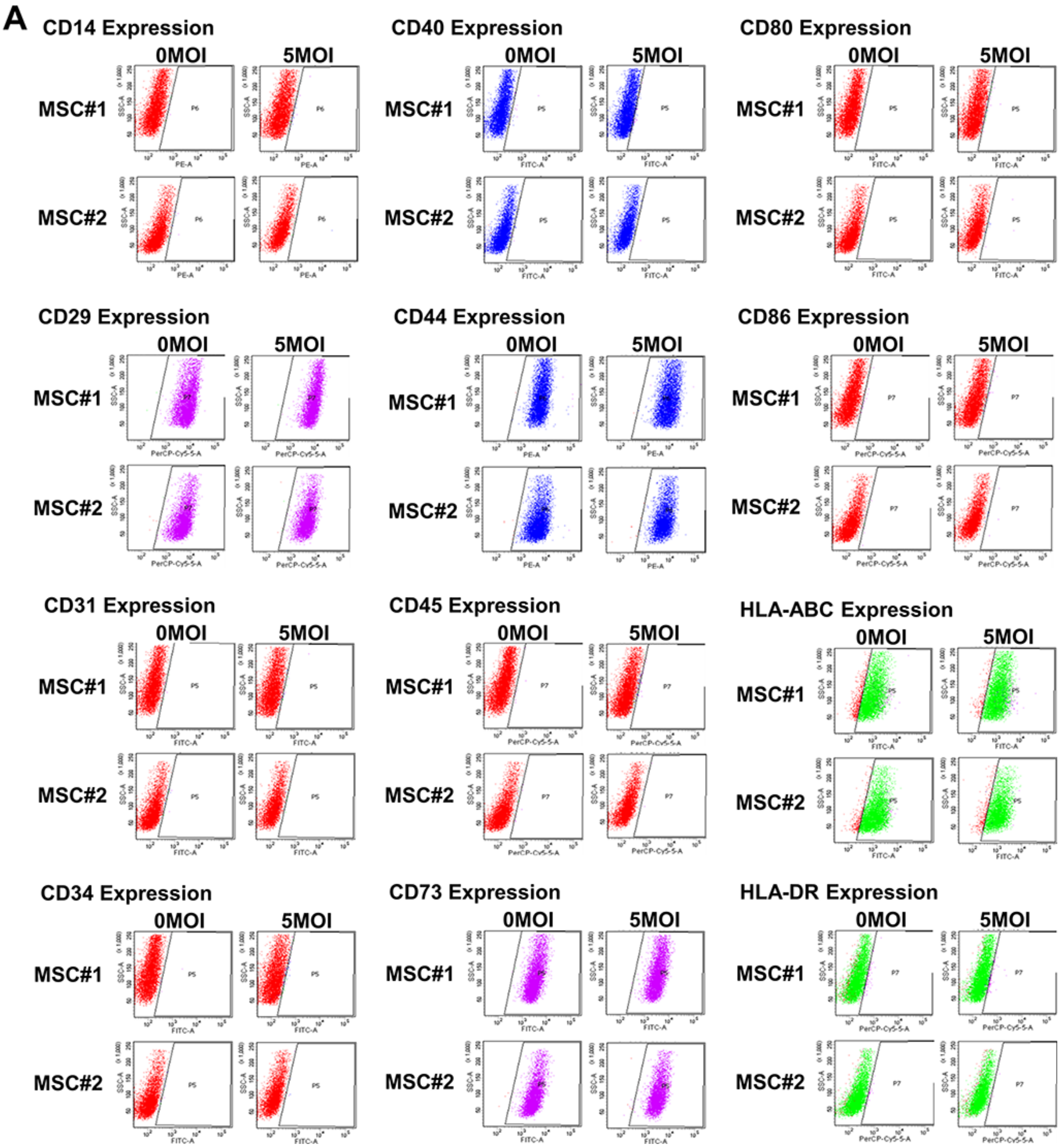

Figure S1. The expression of cell surface markers for MSCs (**A**) Expression of MSC markers and immunogenicity-related surface antigens was analyzed by flow cytometry.

A

	A	B	C	D	E	F	G	H	I	J	K	L
1	POS	POS	NEG	NEG	ALCAM	Angiopoietin-1	B7-H1	bFGF	DKK-1	DKK-3	Fas Ligand	IGF-I
2	POS	POS	NEG	NEG	ALCAM	Angiopoietin-1	B7-H1	bFGF	DKK-1	DKK-3	Fas Ligand	IGF-I
3	IL-α	IL-1β	IL-1RA	IL-8	MMP-9	SCF	SDF-1α	SDF-1β	Serpin A4	Serpin F1	TSP-1	TSP-2
4	IL-α	IL-1β	IL-1RA	IL-8	MMP-9	SCF	SDF-1α	SDF-1β	Serpin A4	Serpin F1	TSP-1	TSP-2
5	TIMP-2	TRAIL	VEGF-A	VEGF-C	VEGF-D	TGF-β1	Galectin-1	Galectin-3	Galectin-9	HGF	IL-6	IL-10
6	TIMP-2	TRAIL	VEGF-A	VEGF-C	VEGF-D	TGF-β1	Galectin-1	Galectin-3	Galectin-9	HGF	IL-6	IL-10
7	M-CSF	LIF	IL-18	IL-33								POS
8	M-CSF	LIF	IL-18	IL-33								POS

B

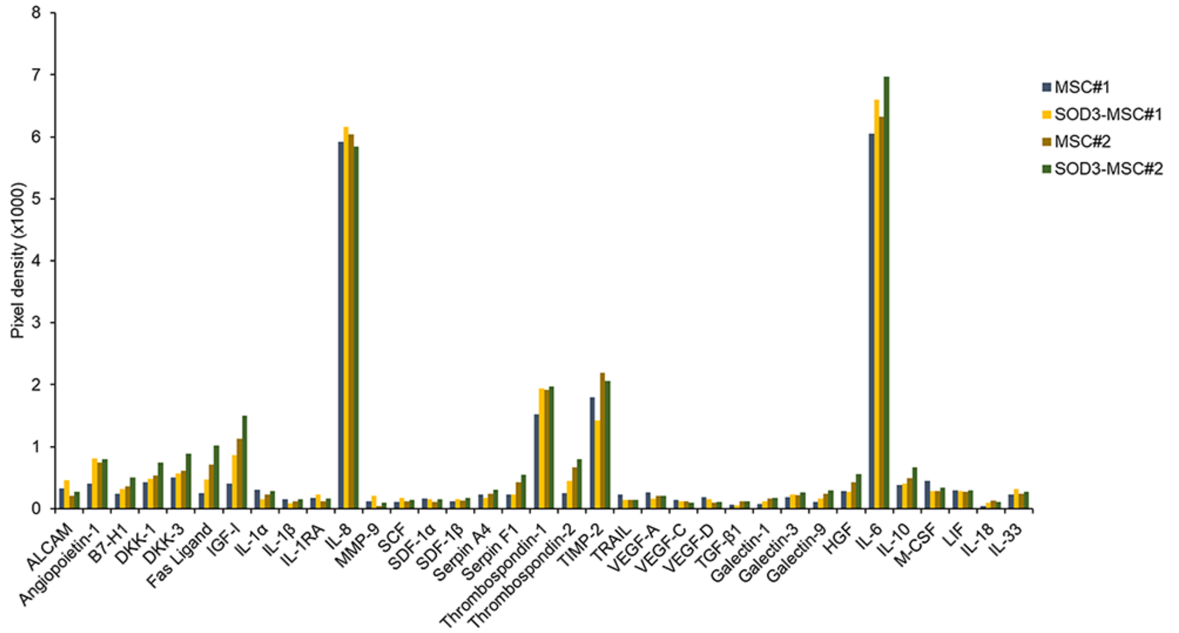

Figure S2. The secretion of soluble factors involved in the immunomodulation and migration in conditioned medium of MSCs (**A**) The table showing the positions of cytokines. (**B**) Quantification of the pixels for each identified cytokine in non-transduced and transduced MSC#1, MSC#2 CM. Data are normalized after background subtraction.

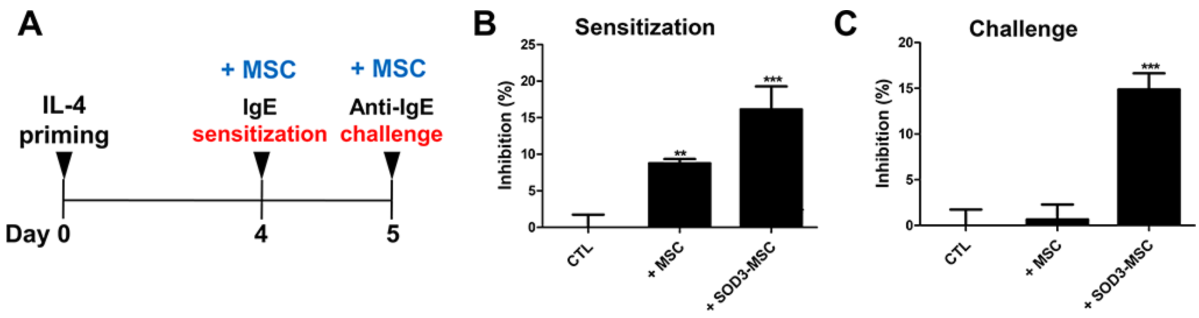

Figure S3. Mast cell degranulation protocol and effect of MSCs on degranulation. (**A**) The timeline showing the process of degranulation induction in LAD-2 cells. (**B, C**) Inhibitory effect of MSCs on LAD2 degranulation was determined by measuring β-hex. Results are shown as mean ± SD. *P < .05, **P < .01, ***P < .001.

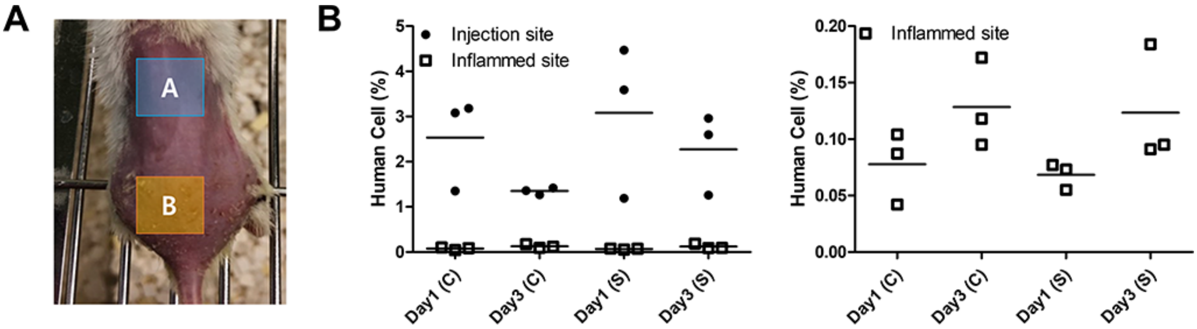

Figure S4. Migratory ability of SOD3-MSCs *in vivo*. (**A**) A photo showing the injection site 'A' and inflamed site 'B'. (**B**) Mice were injected with MSCs or SOD3-MSCs. The % amount of MSCs and SOD3-MSCs in skin samples were measured by quantifying human ALU gene. The amount of MSCs and SOD3-MSCs in the injection site and inflamed site was compared at day 1 and 3.

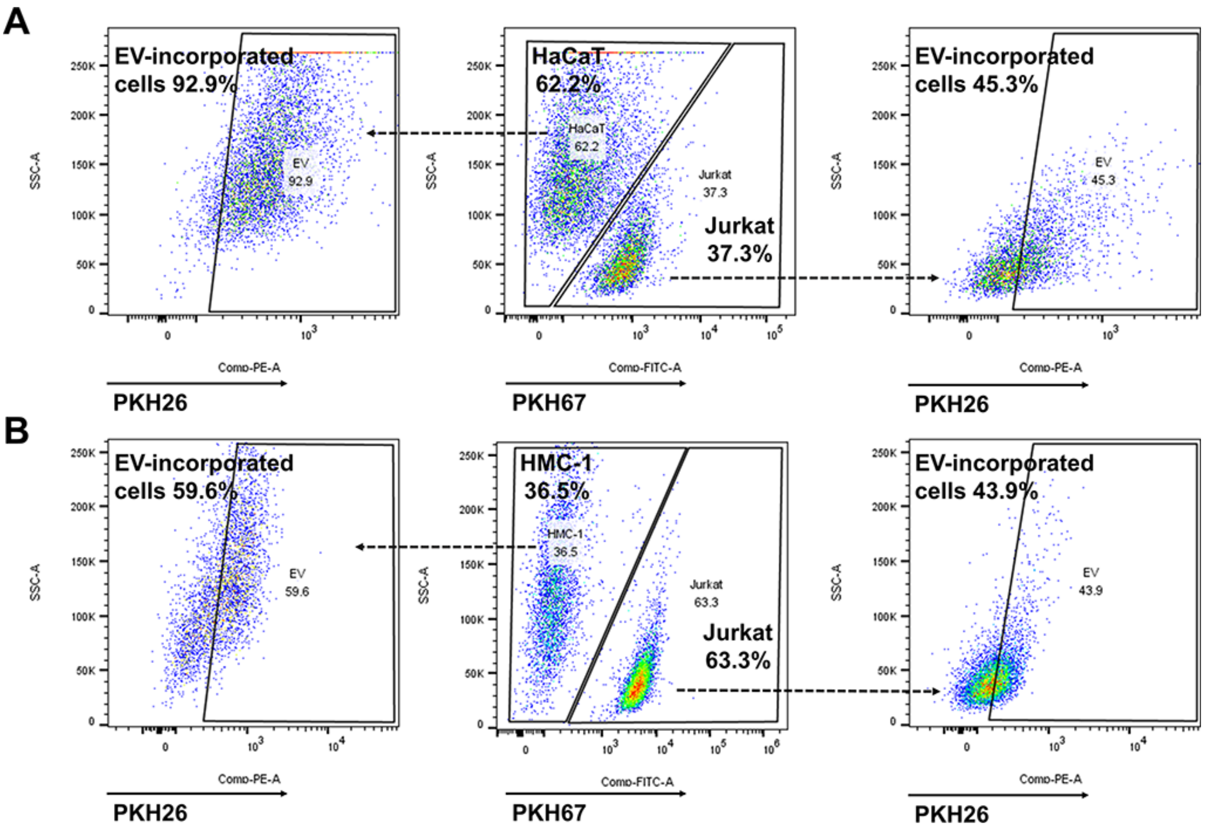

Figure S5. Evaluation of EV uptake efficiency into mast cells, keratinocytes and Jurkat cells. (**A**) EV uptake efficiency into HaCaT cells and Jurkat cells were analyzed by flow cytometry. Unlabeled HaCaT cells and PKH67-labeled Jurkat cells were treated with PKH26 stained EVs. (**B**) EV uptake efficiency into HMC-1 cells and Jurkat cells were analyzed by flow cytometry. Unlabeled HMC-1 cells and PKH67-labeled Jurkat cells were treated with PKH26 stained EVs.

Supplementary Materials and Methods

Internalization of EVs – Related to Figure S5

hUCB-MSCs derived EVs were labeled with PKH26 red fluorescent cell linker kit (Sigma, Saint Louis, MO, USA) according to the manufacturer's instruction. Labeled exosomes were treated into co-cultured cells unlabeled HaCaT and PKH67 (Sigma)-labeled T cell or unlabeled HMC-1 and PKH67-labeled T cell for 24 h. Internalization of EVs were detected by BD FACS CantoII flow cytometer (BD), and data were analyzed with FlowJo software (BD).
